# Combining Chemical Cross-linking and Mass Spectrometry of Intact Protein Complexes to Study the Architecture of Multi-subunit Protein Assemblies

**DOI:** 10.3791/56747

**Published:** 2017-11-28

**Authors:** Caroline Haupt, Tommy Hofmann, Sabine Wittig, Susann Kostmann, Argyris Politis, Carla Schmidt

**Affiliations:** ^1^Interdisciplinary research center HALOmem, Martin Luther University Halle-Wittenberg; ^2^Department of Chemistry, Kings College London

**Keywords:** Biochemistry, Issue 129, Mass spectrometry, cross-linking, native mass spectrometry, protein complexes, protein interactions, protein stoichiometry, protein network, data analysis

## Abstract

Proteins interact with their ligands to form active and dynamic assemblies which carry out various cellular functions. Elucidating these interactions is therefore fundamental for the understanding of cellular processes. However, many protein complexes are dynamic assemblies and are not accessible by conventional structural techniques. Mass spectrometry contributes to the structural investigation of these assemblies, and particularly the combination of various mass spectrometric techniques delivers valuable insights into their structural arrangement.

In this article, we describe the application and combination of two complementary mass spectrometric techniques, namely chemical cross-linking coupled with mass spectrometry and native mass spectrometry. Chemical cross-linking involves the covalent linkage of amino acids in close proximity by using chemical reagents. After digestion with proteases, cross-linked di-peptides are identified by mass spectrometry and protein interactions sites are uncovered. Native mass spectrometry on the other hand is the analysis of intact protein assemblies in the gas phase of a mass spectrometer. It reveals protein stoichiometries as well as protein and ligand interactions. Both techniques therefore deliver complementary information on the structure of protein-ligand assemblies and their combination proved powerful in previous studies.

**Figure:**
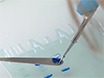


## Introduction

The structural investigation of protein assemblies has become particularly important for the understanding of cellular processes. Consequently, many, techniques have been developed and improved in structural biology^1^. However, these techniques are sometimes limited in their application due to size, flexibility, or heterogeneity of the protein complexes under investigation. Mass spectrometry can deal with these challenges and, therefore, emerged as a powerful tool in structural biology^2^,^3^,^4^,^5^. The greatest advantage of mass spectrometry, however, is the ability to unambiguously identify proteins even in complex and heterogeneous mixtures^6^. To this end, proteins are usually digested with endoproteinases and the resulting peptide mixture is separated by liquid chromatography and directly eluted into the mass spectrometer. Peptide masses are subsequently determined and precursor ions are selected for further fragmentation of the peptides. Proteins are then identified by searching peptide and corresponding fragment masses against a known database. This procedure not only allows the identification of peptides/proteins but also their post-translational modifications which cause a mass shift of the precursor peptides and the fragment ions carrying the modification^7^. Some of the many techniques in structural mass spectrometry are based on this principle^4^,^8^. For instance, labeling techniques such as hydrogen deuterium exchange^9^,^10^, chemical labeling strategies^11^,^12^, or hydroxyl radical foot printing^13^,^14^, give insights into the surface accessibility of the proteins under certain conditions.

Another technique is (chemical) cross-linking involving the covalent linkage of amino acids in close proximity through their functional groups. For this, chemical reagents, UV-activatable reagents, or amino acids are employed^15^,^16^. After cross-linking, the proteins are usually hydrolyzed with proteases and cross-linked di-peptides are analyzed by liquid chromatography-coupled mass spectrometry. The identification of cross-linking products by database searching, however, requires the use of specialized software which concatenate peptide sequences of various proteins and disparate regions^17^,^18^,^19^. The use of chemical cross-linkers has the advantage that it can be employed to almost every protein complex of interest and does not require incorporation of UV-activatable amino acids, which can only be achieved when expressing the protein of interest in host cells. As such, cross-linking is a versatile tool and was successfully employed in many structural studies of even large protein assemblies^20^.

Mass spectrometry of intact protein complexes (sometimes called 'native' mass spectrometry), on the other hand, involves the analysis of intact proteins and protein complexes without hydrolysis into peptides. It reveals composition, heterogeneity, stoichiometry, topology, and subunit interactions of protein complexes^21^,^22^. In combination with ion mobility, native mass spectrometry further allows determination of their conformation^23^,^24^. This makes it a powerful tool for the structural investigation of protein complexes that are difficult to assess by conventional structural techniques. However, native mass spectrometry requires analysis buffers which maintain non-covalent protein interactions during electrospray ionization. This is usually achieved by using aqueous, volatile buffers such as ammonium acetate^25^. In addition, instrument modifications are necessary to prevent dissociation during transmission into the gas phase of the mass spectrometer^26^. Applied in this way, many (large) protein complexes were analyzed. Impressive examples are the studies of intact ribosomes^27^, ATP synthases^28^, or viruses^29^.

The combination of native mass spectrometry and cross-linking proved particularly successful in previous studies. For instance, the stoichiometries of chaperone complexes, including an unexpected Hsp70 dimer, could be obtained from mass spectrometry experiments of the intact protein complexes, while chemical cross-linking revealed the arrangements of the proteins in the assemblies^30^,^31^. In a different study, the effects of post-translational modifications on an intact ATP synthase complex were studied. Native mass spectrometry provided insights into the protein complex stability in the presence or absence of phosphorylation or acetylation^32^,^33^. A comparative cross-linking strategy^34^ then revealed conformational changes of the protein complex under the different conditions.

Here, we provide the protocols for protein identification by mass spectrometry, (chemical) cross-linking, and native mass spectrometry, including data analysis and interpretation (Figure 1). The combination of complementary results obtained from these methods with the help of two well-characterized protein complexes is demonstrated^35^. Our protocol can be applied to any protein assembly which can be purified at certain purity and concentration. The approach is in some cases limited by data analysis of cross-linking data, *i.e.*, the size of the database employed, which determines the required search space and time. In addition, manual validation of identified cross-links is often required and further reduces the output. Native mass spectrometry is mostly limited by the sample quality, *e.g.*, buffers and adducts used during purification and the possibility to exchange them by aqueous and volatile buffers. However, protein complexes purified for structural analysis usually have the quality required for successful analysis with our protocols.

## Protocol

### 1. Purification of Protein Complexes

1. Prepare protein complex according to optimized standard protocols. NOTE: The protocol is demonstrated here with the RvB1/B2 complex from *Chaetomium thermophilum* and the carbamoyl phosphate synthase (CPS) from *Escherichia coli*. RvB1/B2 and CPS were purified as described^36^,^37^. Every protein complex requires an individual purification protocol. Adjust the protocol accordingly. Amine-free buffers such as phosphate-buffered saline (PBS) or 2-[4-(2-hydroxyethyl)piperazin-1-yl]ethanesulfonic acid (HEPES) should be used for chemical cross-linking. Replace the buffer during purification if possible. CAUTION: Do not apply any methods or reagents that disturb the native assembly of the protein complex.

### 2. Mass Spectrometry-based Protein Identification

1. **Gel electrophoresis** NOTE: There are different gel systems available and every laboratory uses its own setup. Adjust the conditions according to the gel system. Wear gloves and lab coat throughout the protocol as keratins are among the most common contaminants in mass spectrometric analysis.
  1. Apply 7.5 µL 4x sample buffer and 3 µL 10x reducing agent (final concentration 50 mM dithiothreitol (DTT)) to 20 µL protein sample. Use 10 µM CPS or 2.5 µM RvB1/B2. Spin down for 1 min at 16,200 × g and heat for 10 min at 70 °C. NOTE: When using a different gel system adjust the required volumes and heating temperature.
  2. Prepare a 4 - 12% gradient gel for electrophoresis. Dilute the running buffer 20 times with water and fill the electrophoresis chamber. Use 2-(N-morpholino)ethanesulfonic acid (MES) buffer for proteins of smaller molecular weight (2 - 200 kDa) and 3-(N-morpholino)propanesulfonic acid (MOPS) buffer for larger proteins (14 - 200 kDa). Add 0.5 mL antioxidant to the inner chamber. NOTE: When using a different gel system prepare running buffer and the gel according to manufacturer's protocols.
  3. Load a suitable protein marker (pre-stained, unstained, different molecular sizes) in the first cavity and load the protein samples into the remaining cavities.
  4. Separate proteins for 35 min (MES) or 50 min (MOPS) at 200 V. NOTE: Adjust the electrophoresis times and voltage for different gel systems.
  5. To stain the protein bands, transfer the gel into a gel staining box and cover the gel with a water-based Coomassie staining solution. Incubate overnight and at room temperature on a horizontal gel shaker.
  6. Destain the gel by replacing the staining solution with water. Repeat this step several times (approximately 3-5 times) until the gel background appears clear.
2. **In-gel digestion** NOTE: To avoid contamination, use HPLC grade solvents throughout the digestion protocol and for all following steps (*i.e.*, mass spectrometric analysis). Prepare all solutions prior to use and filtrate water and ammonium bicarbonate.
  1. Cut the protein bands that are visualized by blue Coomassie stain from the gel using a scalpel. Carefully cut the protein bands into small pieces of approximately 1 mm × 1 mm. Rinse the scalpel with water between different protein bands. Wash the gel bands with water and acetonitrile (ACN).
  2. Reduce disulphide bonds with DTT, alkylate cysteine residues with iodoacetamide, and digest proteins with trypsin as previously described^38^; usually an enzyme:protein ratio of 1:20 up to 1:100 is used. Use 100 mM ammonium bicarbonate during the digestion protocol.
  3. Extract peptides in two steps.
    1. First incubate the gel pieces with ammonium bicarbonate and ACN, and collect the peptide-containing supernatant. Second, incubate the gel pieces with 5% (v/v) formic acid and ACN. Incubate for 15 min at each step. Combine both supernatants. Dry the extracted peptides by evaporating the solvents in a vacuum centrifuge. NOTE: Dried peptides can be stored at -20 °C for several months.
3. **Liquid chromatography-coupled mass spectrometry**
  1. Dissolve the dried peptides in 2% ACN/0.1% formic acid. Dissolve the peptides in a sonication bath for 2-3 min and spin down in a centrifuge at 16,200 x g for 30 min. Transfer the samples into autosampler vials. NOTE: Adjust the volume according to the protein amount. For well-stained Coomassie bands we use 20 µL.
  2. Inject 5 µL of the sample to the nano-LC-MS/MS system using the autosampler. Load the peptide mixture onto a reversed-phase C18 pre-column (C18, 150 µm I.D., 2 cm, 5 µm pore size) to desalt and concentrate the peptides online.
  3. Use 0.1% (v/v) formic acid as mobile phase A and 80% ACN/0.1% (v/v) formic acid as mobile phase B. Separate the peptides on a reversed-phase C18 analytical column (C18, 75 µm I.D., 50 cm, 3 µm pore size) using a gradient of 4-80% B (containing 0.1% formic acid) at 300 nL/min over 65 min. NOTE: A nanospray ion source is used to transfer eluted peptides into the mass spectrometer.
  4. Use (typical) MS conditions: spray voltage of 1.6 kV; capillary temperature of 250 °C; normalized collision energy of 30. Operate the mass spectrometer in data-dependent mode.
  5. Acquire the MS spectra in the mass analyzer (*e.g.*, orbitrap) (m/z 350−1,600) with a resolution of 70,000 and an automatic gain control target of 3 x 10^6^. Select 20 of the most intense ions for HCD fragmentation at an automatic gain control target of 1 x 10^5^. Dynamically exclude previously selected ions for 30 s. Exclude singly charged ions as well as ions with unrecognized charge state. NOTE: Internal calibration of the mass spectra was performed using the lock mass option^39^.
4. **Database searching** NOTE: There is different software available for database searching. MaxQuant^40^, for instance, is freely available.
  1. Convert .raw-files into .mgf-files using a pXtract conversion tool (http://pfind.ict.ac.cn/downloads.html).
  2. Perform database search using typical search parameters: Database, swissprot; peptide mass tolerance, 10 ppm; fragment mass tolerance, 0.5 Da; enzyme, trypsin; missed cleavage sites, 2; variable modifications, carbamidomethylation (cysteine) and oxidation (methionine). NOTE: Change the search parameters according to the experimental settings.
  3. Inspect the database search result. Evaluate the protein score, number of peptides identified, peptide scores, and mass accuracy; the sequence coverage returns the protein coverage identified. NOTE: Every search engine has its individual scoring algorithm. Evaluate the peptide scoring system by the quality of the tandem mass spectra observed. The main signals of a good spectrum should show (complete) ion series to verify the identified peptide. Usually the peptide score is probability-based, *i.e.*, the peptide score is a measure for how likely the identified peptide sequence matches the obtained spectrum. The protein score is usually derived from peptide scores and is used to rank a protein in a list of identified proteins.

### 3. Mass Spectrometry of Intact Protein Complexes (Native Mass Spectrometry)

1. **Preparation of gold-coated emitters for electrospray ionization**
  1. Use a micropipette puller to prepare nanoflow emitters from glass capillaries as described previously^25^,^41^. Use borosilicate capillaries with an inner diameter of 0.78 mm. NOTE: By changing the parameters for needle pulling, the tip shape and size can be modified and adjusted for the sample. Capillaries with different inner diameter and wall thickness are available.
  2. Coat the glass capillaries with conductive material (*e.g.*, gold or palladium); the use of a sputter coater generating a gold plasma is common. Follow the manufacturer's instructions to obtain good quality coating. NOTE: The coating should be sufficient enough to obtain a stable spray when applying common capillary voltages (see below).
2. **Sample preparation for native mass spectrometry** NOTE: Salts, detergents, or large amounts of glycerol are not compatible with electrospray ionization. Therefore, the purification buffer is exchanged by a volatile, aqueous buffer. 200 mM ammonium acetate is commonly used. Use size exclusion spin columns or ultrafiltration devices for buffer exchange. In some cases, complex stability or activity might be affected by buffer exchange. Evaluate the mass spectra carefully and check the activity of the complex. Add cofactors or additives to the analysis buffer, if necessary.
  1. Use size exclusion spin columns for fast buffer exchange. Remove the storage buffer by centrifugation at 1,000 x g and 4 °C for 1 min. Discard the flow through. Wash three times by adding 500 µL 200 mM ammonium acetate, followed by centrifugation. Load 20 µL of the protein sample onto the column and centrifuge at 1,000 x g and 4 °C for 4 min. NOTE: The concentration of the protein complex should be 1-10 µM. The procedure can be repeated if non-volatile components still disturb the analysis.
  2. To concentrate the protein sample and exchange the buffer in the same experiment use centrifugal filters. Use a filtration membrane of pore size 50% smaller than the size of the proteins analyzed.
    1. Transfer the protein sample into the filtration device and add 200 mM ammonium acetate. Spin down at 15,000 x g and discard the flow through. Add 200 mM ammonium acetate and repeat the centrifugation. Repeat this step several times. Follow the manufacturer's instructions for centrifugation speed. Perform the centrifugation at 4 °C. **Caution:** Membrane proteins tend to precipitate on the membrane of the filter device.
3. **Mass spectrometric analysis of intact protein complexes** NOTE: There are different types of mass spectrometers from different manufacturers that can be modified for native mass spectrometry, *e.g.*, quadrupole time-of-flight (Q-ToF) mass spectrometers or orbitrap mass spectrometers. The protocol described below was performed on a Q-ToF instrument.
  1. Place a gold-coated capillary in the capillary holder and fill the capillary with 1-4 µL of protein sample. Open the tip of the needle with a tweezer.
  2. Connect the capillary holder with the nano-electrospray source and adjust the capillary position. Position the tip of the capillary at 0.5-1.5 cm to the cone orifice. Use 80-150 L/h nanoflow gas to initiate the spray and adjust the gas flow to maintain a stable spray.
  3. Adjust parameters in the tune-page of the Q-ToF instrument. Typical starting conditions are: capillary voltage, 1.50 kV; cone voltage, 80 V; RF lens 1 energy, 80 V; collision energy, 20 V; aperture 3, 13.6 V. Modify these parameters to obtain good mass spectra. Start the acquisition by clicking the "acquire" button and combine as many scans as possible to obtain a good mass spectrum. NOTE: We recommend combining at least 100 scans.
4. **Tandem mass spectrometry of intact protein complexes**
  1. Acquire the mass spectrum as described above (protocol section 3.3). Choose a precursor ion of a protein complex.
  2. Change from MS into MS/MS mode in the acquisition file. Set the MS/MS selection to the precursor mass.
  3. Start the acquisition at low collision energy. Combine several scans (approximately 20 scans) to verify selection of the correct precursor mass. Increase the collision energy until the protein complex dissociates. To obtain a good mass spectrum combine at least 500 scans. NOTE: Stripped complexes sometimes have low intensity. Combining as many scans as possible might increase the resolution and signal-to-noise ratio.
5. **In-solution dissociation of intact protein complexes** NOTE: To gain additional insight into protein interactions within the protein complexes, in-solution dissociation can be performed.
  1. Prepare the protein sample as described above (section 3.2). Add solvents to the protein sample or change the pH to dissociate intact complexes into sub-complexes. Typical solvents are methanol, isopropanol, and ACN; change the pH of ammonium acetate by the addition of acetic acid or ammonia solution. Acquire mass spectra as described above (sections 3.3 and 3.4).
  2. Vary the amount of solvent or the pH range to generate various sub-complexes. Typically, the amount of solvent is 5-50% (final concentration) and a typical pH range is 4-9. Start with a low concentration of solvent (5%) or slight changes in pH and acquire a mass spectrum (see section 3.3).
  3. Increase the amount of solvent or change the pH stepwise until sub-complexes are generated in solution. Acquire mass spectra to analyze sub-complexes.
6. **Calibrate data** NOTE: Acquired mass spectra are calibrated externally using Cesium iodide (CsI) solution.
  1. Dissolve 100 mg CsI in 1 mL water.
  2. Acquire a mass spectrum of CsI. Vary the collision energy to obtain CsI clusters over the same *m/z* range as the protein complex analyzed above. CAUTION: CsI quickly precipitates at the emitter tip and contaminates the cone. Acquire only as many scans as required to gain a sufficient spectrum. Remove the emitter from the source when finished.
  3. Make a calibration file using the acquired mass spectrum and a CsI reference file.
  4. Apply calibration to acquired mass spectra. CAUTION: Calibration of mass spectra might be a permanent change of the raw data. If non-calibrated spectra are needed, make a back-up copy of the file.
7. **Data processing and analysis** NOTE: There are many freely-available software tools for data analysis of native mass spectra; for instance, Massign^42^ or UniDec^43^. The protocol below describes manual data analysis with the help of instrument software as well as the use of Massign for complex samples. This software is well-suited for the analysis of complex mass spectra. Follow the instructions provided online for use of the program (http://massign.chem.ox.ac.uk/).
  1. For data analysis, smooth spectra by adjusting smoothing parameters. Centroid spectra by adjusting parameters. Calculate complex masses from two adjacent peaks of the protein complex' peak envelope using instrument software tools. CAUTION: Too intensive smoothing might cause data loss (*e.g.*, loss of bound ligands).
  2. For analysis with Massign^42^, generate a peak list for the mass spectrum. Linearize data points of the spectrum and smooth. Use the various software tools to assign protein complexes, calculate complex composition, or simulate complex peak envelopes.

### 4. Chemical cross-linking Coupled with Mass Spectrometry

NOTE: There are numerous cross-linking strategies available. Here, we describe the use of Bis(sulfosuccinimidyl)suberate (BS3), an amine-reactive cross-linker which is commonly used to study protein-protein interactions.

1. Dissolve 1.43 mg BS3 in 100 µL water to prepare a 25 mM stock solution. NOTE: Other reagents like disuccinimidyl suberate (DSS) are not water-soluble and are usually dissolved in dimethyl sulfoxide (DMSO). Some cross-linkers are also available in heavy isotopically-labeled forms. Incorporation of heavy stable isotopes generates peak pairs of cross-linked di-peptides in MS spectra, which helps during data evaluation. When using differentially labeled cross-linkers, stock solutions of both variants are prepared and mixed 1:1. CAUTION: To avoid dissociation of the protein complexes by DMSO, a highly concentrated stock solution is prepared and diluted with water or buffer prior to the cross-linking reaction.
2. Add BS3 to the protein complex. Use varying amounts of BS3 ranging from 0.5-5 mM to identify optimal cross-linker concentration. Incubate the reaction mixtures at 25 °C for 1 h in a thermomixer. Use 4-12% gradient gels and perform SDS-PAGE to evaluate cross-linking results (see Figure 2 for an example). NOTE: Optimal concentration of BS3 is reached when higher molecular weight protein bands, which are not visible in the non-cross-linked control, are obtained during SDS-PAGE while single subunits are still visible (Figure 2).
3. Repeat the cross-linking reaction with the optimal BS3 concentration. Incubate the reaction mixtures at 25 °C for 1 h in a thermomixer. NOTE: Some protein complexes are not stable at room temperature. The cross-linking reaction can also be performed on ice; however, the reaction time needs to be adjusted.
4. **Quench the cross-linking reaction by adding amine buffer (*e.g.*, 50-100 mM Tris buffer, pH 7.5, final concentration) or perform ethanol precipitation to remove any residual cross-linking reagent.**
  1. Add water or buffer to the reaction mixture to reach a final volume of 200 µL. Add 600 µL ice-cold ethanol and 20 µL 3 M sodium acetate, pH 5.3. Mix thoroughly and incubate at -20 °C for 2 h or overnight. NOTE: Alternatively, the proteins can be precipitated at 80 °C for 30 min or in liquid nitrogen.
  2. Spin down at 16,200 x g and 4 °C for 30 min. Carefully remove the supernatant.
  3. Wash the pellet with 1 mL ice-cold 80% (v/v) ethanol. Spin down at 16,200 x g and 4 °C for 30 min. Carefully remove the supernatant. Dry the pellet in a vacuum centrifuge.
5. Perform SDS-PAGE of the cross-linked proteins. Cut the gel bands and digest the protein in-gel as described above (sections 2.1 and 2.2). NOTE: In-solution digestion can also be performed, however, it usually requires additional separation steps (*e.g.*, size exclusion chromatography of the digested peptides).
6. **Perform liquid chromatography-coupled mass spectrometry as described above (section 2.3). As cross-linked peptides are usually low abundant, apply the following variations to the mass spectrometric analysis to increase analytical depth of the cross-linked sample.**
  1. Use longer gradients during liquid chromatography separation (*e.g.*, 90 min instead of 65 min, see above).
  2. Exclude the doubly charged peptides from HCD fragmentation. NOTE: Doubly charged peptides are usually intra-cross-linked peptides ("loop peptides" or "type 1" cross-links).
  3. When using differentially labeled cross-linking reagents, use the "peak picking" option during analysis, *i.e.*, HCD fragmentation is triggered by the presence of peak pairs of defined mass difference in the mass spectra. NOTE: The "peak picking" option might not be available on every mass spectrometer.
7. Use pLink software^18^ for identification of cross-linked di-peptides. Use minimized databases for identification. Typical search parameters are: instrument spectra, HCD; enzyme, trypsin; max. missed cleavage sites, 3; variable modifications, oxidation (methionine) and carbamidomethylation (cysteine); cross-linker, BS3; min. peptide length, 4; max. peptide length, 100; min. peptide mass, 400 Da; max. peptide mass, 10,000 Da; FDR, 1%. NOTE: When using differentially labeled cross-linkers, the mass increase caused by the linker needs to be configured. There is also other commonly used software for cross-link identification, *e.g.*, xQuest^17^, MassMatrix^19^, or XlinkX^15^.
8. Evaluate the database search results by the quality of fragmentation spectra. Acceptable spectra of cross-links should show ion series of both peptides (at least 4 adjacent ions) at a reasonable signal-to-noise ratio. NOTE: When using differentially labeled cross-linking reagents, peak pairs in MS spectra can be used as an additional quality control.
9. If required, visualize the cross-linking results in protein interaction networks using software tools (*e.g.*, XVis, XiNET). Use bar plots or circular plots for visualization of protein interactions. NOTE: Both software tools are freely available on a web server. Follow detailed instructions on the respective websites (*https://xvis.genzentrum.lmu.de/* and http://crosslinkviewer.org/).

## Representative Results

The structural analysis of proteins and the complexes they form is fundamental for understanding their function. Mass spectrometry considerably contributes to the structural investigation in that it can be applied to almost every complex of interest irrespective of size or sample heterogeneity. We exemplify the protocol by using two well-characterized protein complexes; first, the hetero-dodecamer RvB1/B2 from *C. thermophilum* and second, the hetero-octameric CPS complex from *E. coli*.

First, we identified the protein components of the two complexes. For this, the proteins were separated by SDS-PAGE (Figure 2) and gel bands were cut from the gel. After in-gel digestion of the proteins, the peptide mixture was analyzed by liquid chromatography-coupled mass spectrometry and peptide and fragment masses were subjected to database searching. Following this workflow, we identified all protein subunits of the two complexes with high confidence, *i.e.*, a high number of peptides with reasonable peptide scores was observed yielding high sequence coverage for all protein subunits (**Table 1**).

We then analyzed the intact RvB1/B2 complex by native mass spectrometry (Figure 3A). The mass spectrum revealed two species, one at approximately 8,000 *m/z* and another species at approximately 11,000-12,000 *m/z*. The calculated masses for these species correspond to the hexameric (RvB1)_3_(RvB2)_3_ ring (approximately 310 kDa) and the dodecameric double-ring (RvB1)_6_(RvB2)_6_ (approximately 620 kDa). Both peak series show two populations; these originate from a mixture of His-tagged and untagged RvB2 subunits in the complexes. A crystal structure for the RvB1/B2 complex was previously obtained^36^ and shows the arrangement of the double-ring (Figure 3B). The native mass spectrum therefore confirms the stoichiometry of the intact dodecamer and furthermore reveals a stable sub-complex. In addition, co-existing populations are identified.

To identify protein interaction sites in the RvB1/B2 complex, we chemically cross-linked the purified complex with BS3 cross-linker. We first titrated the amount of BS3 during the cross-linking reaction to determine the optimal concentration. BS3 is amine-specific and covalently links lysine side chains as well as the N-termini of proteins. The cross-linking reaction was followed by SDS-PAGE (Figure 2A). The non-cross-linked complex showed both RvB1 and RvB2 subunits. Adding BS3 to the reaction mixture caused covalent linkage of the proteins resulting in protein bands at higher molecular weight. The SDS gel shows that increasing amounts of BS3 yield higher amounts of cross-linked species while non-cross-linked protein subunits are reduced. We then cut the protein bands from the gel and followed the protocol provided above to identify protein interaction sites. An example spectrum of a cross-linked di-peptide is shown (Figure 3C). The spectrum shows y-ion series of both peptides confirming this protein interaction. In total, we obtained 14 protein interactions, including four cross-links between subunits RvB1 and RvB2 and two cross-links between two copies of RvB2 (**Table 2**). The results from BS3 cross-linking are visualized in an interaction network (Figure 3D) showing intra-molecular interactions as well as interactions between different subunits. Intra-molecular cross-links suggest that both RvB1 and RvB2 subunits fold in a way that N- and C-terminal domains are in close proximity. Note, that intra-molecular interactions cannot be distinguished from inter-molecular interactions of the same subunit in this case. Inter-molecular cross-links between the two subunits were also observed. Of these, two inter-molecular cross-links between RvB1 and RvB2 could be visualized in the structure validating the cross-linking approach. The other inter-molecular cross-links are located in flexible loops which are not included in the crystal structure. We also identified two cross-links in RvB2 containing the same peptide sequences. These cross-links can unambiguously be classified as inter-molecular as they must originate from two copies of the same protein (Figure 3D). Our cross-linking experiments reveal protein interaction sites within the complex but also within the protein subunits providing insights into their structural arrangement that could also be confirmed by the existing crystal structure (Figure 3B).

The second protein complex that we studied was CPS. The native mass spectrum (Figure 4A) revealed three protein complexes between 6,000 and 12,000 *m/z*. The largest complex of 640 kDa corresponds to the intact hetero-octamer. The smaller complexes represent two sub-complexes; the dimer of the small and large CPS subunits (160 kDa) and a hetero-tetramer containing two copies of each subunit (320 kDa). These sub-complexes deliver first insights into the protein assembly; *i.e.*, the large and small subunits are in direct contact (as revealed by the hetero-dimer) and the tetramer might be a product of two dimers. To gain more information on the structural arrangement in the intact CPS complex, we performed tandem mass spectrometry (MS/MS) of the hetero-octamer and the hetero-tetramer. In both cases, the small subunit dissociated from the precursor suggesting that the small subunit is located in the periphery of the assembly (Figure 4B). Indeed, the small subunit is peripheral in the available crystal structure (Figure 4C)^44^.

Chemical cross-linking using the BS3 cross-linker was also performed. Using increasing amounts, the covalent linkage of the CPS subunits was enhanced. After digestion of the proteins and analysis of the peptides as described above, many protein interactions within the large subunit and one cross-link in the small subunit were obtained (**Table 2**). In addition, similar to the RvB1/B2 complex, we found two inter-molecular cross-links between two copies of the large CPS subunit. These cross-links place the two large subunits facing each other at their C-terminal sides. In a previous study, combining structural mass spectrometry and computational modeling^35^, we identified three additional interactions in the large subunit which most likely originate from the interface of two copies of the large subunit validated by the crystal structure and the obtained model (Figure 4C and **Table 2**). These interactions allow the arrangement of the CPS core complex consisting of four large subunits. However, no inter-subunit cross-links between the small and the large subunits were observed. By inspecting the available crystal structure (Figure 4C), it becomes apparent that the interaction surface between the tetrameric core of the complex, consisting of the large subunit, and the peripheral small subunits is very small, which might explain the absence of inter-subunit interactions. This is confirmed by native mass spectrometry which showed that the small subunit readily dissociates from the intact complexes most likely due to a small binding interface. Nonetheless, protein interactions in the CPS complex combined from chemical cross-linking and native mass spectrometry allow deducing their structural arrangement (Figure 4D).

Taken together, the combination of native mass spectrometry and chemical cross-linking coupled with mass spectrometric identification of cross-linked peptides, allows reconstitution of the structural arrangement of both example complexes. While chemical cross-linking revealed arrangement of the protein subunits, for instance the interactions between RvB1 and RvB2 or within the tetrameric core of CPS, native mass spectrometry delivered protein stoichiometries of the intact complexes and prevalent subcomplexes. In the case of CPS, for which no inter-molecular interactions between the two subunits could be observed by chemical cross-linking, native mass spectrometry suggests that each large subunit interacts with one small subunit (Figure 4D). Tandem mass spectrometry proposed the peripheral location of the small subunit in the complex and a small interface between both subunits.

**Figure:**
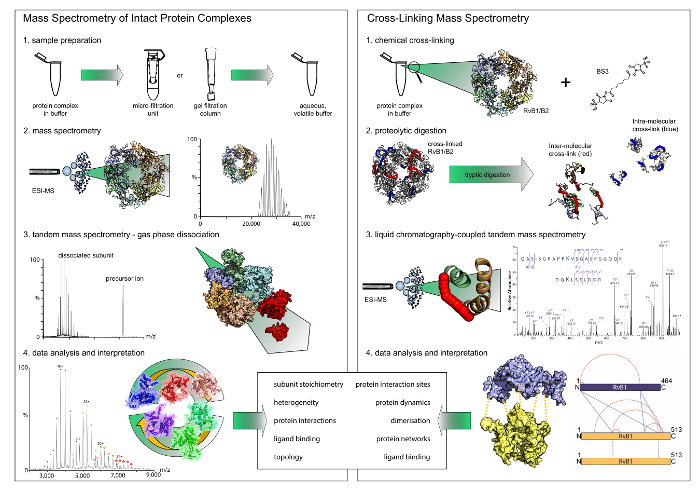

**Figure 1: Workflow of native mass spectrometry and cross-linking.** Both techniques deliver complementary results. While native mass spectrometry reveals stoichiometries and interaction modules, cross-linking gives insights into the protein interaction sites within the complexes. Note that chemical cross-linking only reveals binary interactions. (**A**) The first step in native mass spectrometry is buffer exchange to a volatile and aqueous buffer using filter units or gel filtration columns. Mass spectrometry of the intact protein complexes then reveals their stoichiometry. In tandem mass spectrometry experiments, peripheral subunits are dissociated. (**B**) For chemical cross-linking, the protein complex is incubated with a cross-linking reagent. The cross-linked proteins are then digested into peptides which are subsequently analyzed by liquid chromatography-coupled mass spectrometry. Please click here to view a larger version of this figure.

**Figure:**
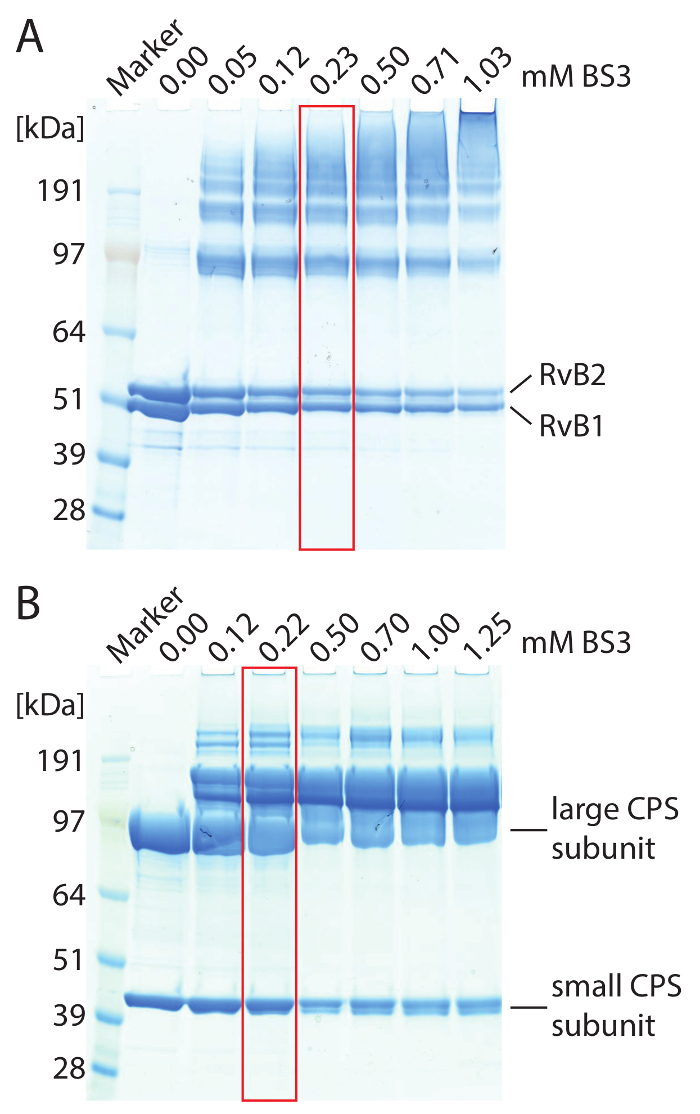

**Figure 2: SDS-PAGE of cross-linked RvB1/B2 (A) and CPS (B) complexes.** (**A**) 2.5 µM RvB1/B2 were loaded per gel lane. The concentration of BS3 was varied. Non-cross-linked RvB1/B2 shows the two protein subunits at approximately 50 kDa. Addition of BS3 caused covalent linkage of the protein subunits resulting in protein bands at higher molecular weight. The amount of cross-linked species is increased with higher BS3 concentrations. Optimal cross-linking conditions are highlighted (red). (**B**) 10 µM CPS were loaded per gel lane. The large (90 kDa) and small (40 kDa) CPS subunits are obtained. Addition of BS3 caused covalent linkage of the protein subunits resulting in protein bands at higher molecular weight. Optimal cross-linking conditions are highlighted (red). Please click here to view a larger version of this figure.

**Figure:**
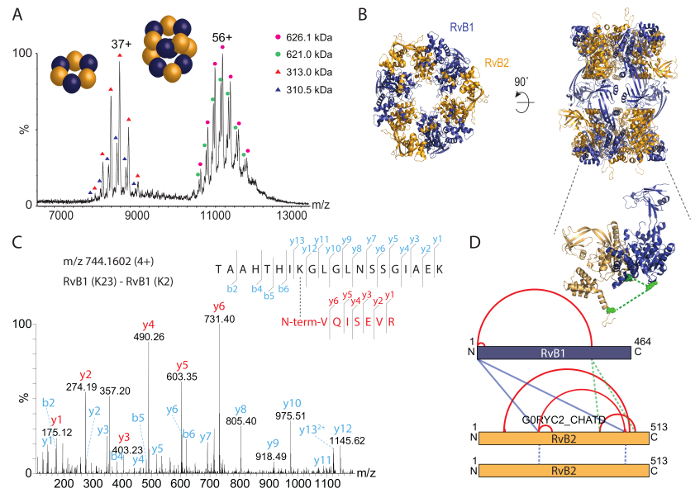

**Figure 3: Native mass spectrometry and chemical cross-linking of RvB1/B2 complex.** (**A**) The native mass spectrum reveals two species of RvB1/B2; the intact dodecamer (*i.e.*, (RvB1)_6_(RvB2)_6_) at approximately 11,000 to 12,000 *m/z* and the hexameric ring (RvB1)_3_(RvB2)_3 _at approximately 8,000 *m/z*. Both species show two populations resulting from His-tagged and untagged RvB2. The spectrum has been modified from^35^. (**B**) The crystal structure of RvB1/B2 is shown (PDB ID 4WVY). Alternating RvB1 and RvB2 subunits form two hexameric rings. (**C**) Fragmentation spectrum of a cross-linked di-peptide. The N-terminus of RvB1 was cross-linked with K23 of RvB1. y-ion series were obtained for both peptides (red and cyan). (**D**) Intra- and inter-protein interactions obtained in the RvB1/B2 complex. Intra-cross-links are shown in red, inter-cross-links are shown in blue. The insert shows two inter-molecular cross-links between RvB1 and RvB2 subunits which could be visualized in the crystal structure (green, insert). Interactions that originate from two RvB2 copies are shown as blue dotted lines. Please click here to view a larger version of this figure.

**Figure:**
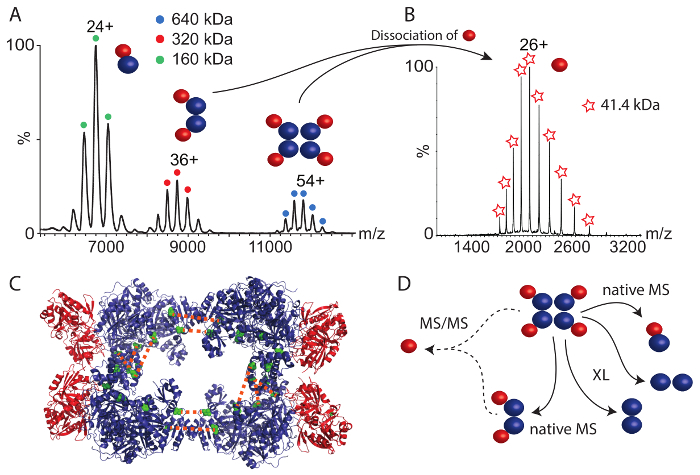

**Figure 4: Native mass spectrometry and chemical cross-linking of CPS.** (**A**) The native mass spectrum of CPS shows three complexes. The hetero-dimer (160 kDa), hetero-tetramer (320 kDa) and the hetero-octamer (640 kDa). The spectrum has been modified from^35^. (**B**) Tandem mass spectrometry of the tetrameric and octameric CPS complex revealed dissociation of the small CPS subunit. (**C**) The crystal structure of CPS is shown (PDB ID 1BXR). The large subunits form a tetrameric core and the small subunits are located in the periphery of the complex. Inter-molecular cross-links between two copies of the large subunit are shown (green). (**D**) Interactions of the large and small CPS subunits. Native mass spectrometry revealed subcomplexes and suggests a peripheral location of the small subunit. Chemical cross-links indicate arrangements in the tetrameric core of CPS. Please click here to view a larger version of this figure.

**Table 1: Database search results. **The proteins were identified by liquid chromatography-coupled mass spectrometry and database searching. The protein names, accession number, and description as well as protein mass are given. The protein score, number of observed spectra per protein, and the number of observed peptide sequences are listed. The five peptides with highest Mascot peptides scores are listed for each protein subunit. Please click here to download this file.

**Table 2: Cross-links observed in RvB1/B2 and CPS.** The subunits of the complexes and the cross-linked residues are given. The type of cross-link (intra- or inter-molecular) was revealed from overlapping peptide sequences or a previous study^35^. Please click here to download this file.

## Discussion

Protocols are provided for mass spectrometry-based structural analysis of multi-subunit protein complexes. The two techniques, described in the protocol, mostly deliver complementary results and are well-suited to gain insights into the structural arrangements within protein (-ligand) complexes which are difficult to study by conventional structural techniques. Native mass spectrometry delivers insights into protein stoichiometries as well as protein interactions by analyzing subcomplexes and stable interaction modules. Cross-linking, on the other hand, yields information on direct contact sites. Depending on the cross-linker used, a certain flexibility can or should be included in the analysis.

The provided protocols are in general easy to perform and not time-consuming. The entire protocol can be executed within a week and can be applied to almost all protein complexes, although, a certain amount of the protein complex is required for successful analysis. Sample preparation is simple and does not require specifically purified protein complexes. However, one common pitfall is contamination of the sample during sample preparation for mass spectrometry-based protein identification. These contaminations in most cases include keratins which originate from dust, skin, or hair. Therefore, extra care such as wearing gloves and lab coats, filtrating aqueous buffers, and using high-purity solvents should be taken during sample preparation for mass spectrometry-based protein identification. Other contaminating proteins such as chaperones are usually introduced during protein purification, *e.g.*, when using affinity tags. In these cases, protein purification should be improved, for instance by increasing washing steps. In any case, protein contaminations in the sample are easily identified during the database search by omitting the taxonomy filter (*i.e.*, searching against proteins from all species). If only a few peptides are observed (*i.e.*, a low protein coverage could be obtained), even though sufficient sample is available, it might be necessary to use a different proteinase during digestion. In general Trypsin yields a sufficient number of peptides; however, in some cases such as membrane proteins or membrane domains of proteins, the number of tryptic cleavage sites is reduced and other enzymes targeting hydrophobic amino acids are a better choice.

In terms of instrumentation, a particularly modified instrument is required for native mass spectrometry which maintains non-covalent interactions during transfer into the gas-phase. Several instrument types have been introduced, including Q-ToF and orbitrap instruments. While modified Q-ToF mass spectrometers are commercially available for native mass spectrometry since several years, the latter were only recently introduced and in most cases require specialized modification^45^. However, the application of high-resolution instruments allowed studying binding of multiple ligands and their quantification^46^,^47^ and is promising for future applications.

To identify cross-linked di-peptides by liquid chromatography-coupled mass spectrometry, standard procedures with few modifications can be applied. However, the database search is the limiting factor as specialized software can rarely deal with large databases, and reduced databases containing the protein subunits of the complexes are required. Recent studies used mass spectrometry-cleavable cross-linkers targeting protein interactions in entire cell lysates^48^,^49^. The use of chemical cross-linkers that fragment in tandem mass spectrometry experiments mostly yields linear peptides (modified by the cross-linker), which can be identified by further fragmentation and database searching of linear peptides, and this reduces search time and computational search space. However, to perform these experiments, an ion trap mass analyzer or a hybrid mass spectrometer with an ion trap is required. In general, as false-positives are an important issue, mass spectra of cross-linked peptides are often validated manually by the quality of their fragment spectra which extends data analysis time enormously. Developing robust scoring systems that can be applied without further validation steps are therefore potential future applications. One way to improve data analysis and to reduce the number of false-positives was the introduction of false discovery rate calculations and their application to cross-linking data sets^50^.

In general, the techniques described here can be complemented with further mass spectrometry techniques (*e.g.*, covalent labeling) to increase the output from the analysis. Other modifications and improvements of the protocols can be easily implemented. As such comparative cross-linking^34^ unravels conformational changes in the protein assembly. Further developments in native mass spectrometry nowadays allow the analysis of membrane proteins^51^,^52^ and their interactions with lipids^28^,^52^,^53^,^54^. New developments of high-resolution mass spectrometers for native mass spectrometry have extended the application and ligand binding, *e.g.*, binding of lipids to membrane proteins, can now be included in the analysis^45^,^46^. In combination with computational modeling approaches, these techniques can deliver structural models of varying resolution^55^. If no crystal structures are available for the intact complex or single subunits, mass spectrometry can deliver first insights into protein interactions and the topology of the unknown complex. Depending on the techniques used and the results obtained, low-resolution models of the unknown complex can be obtained^56^,^57^,^58^. If crystal structures or homology models are available, the structural information received from mass spectrometry can yield even near-native models^59^.

In comparison with other structural techniques, mass spectrometry has the advantage that it requires low sample amounts, it can deal with heterogeneous samples and is applicable to protein complexes of unlimited size. In addition, mass spectrometry allows the investigation of dynamic protein systems. Different populations of the protein or protein complex that exist in solution are usually analyzed together and therefore, unlike with other structural techniques which require selection of certain populations, all conformations are maintained during analysis and are assessable in one experiment. Quantitative cross-linking approaches were recently introduced^34^,^60^,^61^ and are promising for future applications describing conformational changes under different conditions.

## Disclosures

The authors have nothing to disclose.
